# Effect of ischemic compressions versus extracorporeal shockwave therapy on myofascial trigger points: A protocol of a randomized controlled trial

**DOI:** 10.1371/journal.pone.0283337

**Published:** 2023-03-30

**Authors:** Melissa Nahomi Kuroda, Guilherme Thomaz de Aquino Nava, Caroline Baldini Prudencio, Daiane Affonso Paulo, Isadora Peixouto, Maiki Yoshi Moroshima, Mariana de Almeida Lourenço, Caroline Nogueira da Silva, Angélica Mércia Pascon Barbosa, Cristiane Rodrigues Pedroni

**Affiliations:** 1 Department of Physical Education, Institute of Biosciences, São Paulo State University (UNESP), Rio Claro, São Paulo, Brazil; 2 Department of Tocogynecology, Botucatu Medical School, São Paulo State University (UNESP), Botucatu, São Paulo, Brazil; 3 Department of Physiotherapy and Occupational Therapy, Faculty of Philosophy and Sciences, São Paulo State University (UNESP), Marilia, São Paulo, Brazil; 4 Department of Physiotherapy, Educational Foundation of the City of Assis (FEMA), Assis, São Paulo, Brazil; Complutense University of Madrid: Universidad Complutense de Madrid, SPAIN

## Abstract

**Introduction:**

The myofascial trigger points (MTrPs) are hyperirritable nodules present in a tight muscle band. Among the symptoms, pain is one of the most common, but the individuals may have other sensory, motor, and autonomic changes. Athletes can have MTrPs more intensely due to the high physical and emotional demand. There are a variety of treatments, but not all have strong or moderate evidence of their effectiveness. Thus, the aim of this study is to compare the effects of ischemic compression (IC) and extracorporeal shockwave therapy (ESWT) on pressure pain threshold immediately after the intervention and after 48h.

**Methods:**

This randomized clinical trial was registered in the Brazilian Registry of Clinical Trial (RBR-6wryhb9) and was approved by the Research Ethics Committee (CAAE 46682921.9.0000.5406). Forty participants will be randomized to receive IC or ESWT treatment once in each MTrPs. The protocol will consist of evaluations before (T0), immediate after (T1), and after forty-eight hours (T2) of the intervention. The primary outcome will be pressure pain threshold and the secondary outcomes will be jump height, muscle strength, dorsiflexion range of motion (ROM), the correlation between MTrPs and temperature and participant’s satisfaction.

**Conclusions:**

The IC and ESWT have been shown to be efficient in decreasing pain, however, the studies that compare the efficiency of these two treatments are limited in the literature, mainly in the muscles of the lower limbs, which are of great importance and are commonly injured. This study will provide evidence of the IC and ESWT in the triceps surae muscles, assisting in a better treatment for the individual with MTrPs.

## Background

Myofascial Pain Syndrome is a musculoskeletal disorder whose main characteristic is the appearance of myofascial trigger points (MTrPs) in muscle regions [1]. Most of the population has had or will have myofascial pain due to MTrPs [1, 2]. The prevalence of MTrPs is independent of gender and age, however, it is more frequent among young adults aged 27 to 50 years and occurs intensely in athletes, due to the high physical and emotional demand, with the triceps surae muscle being one of the most affected [3–6].

MTrPs are hyper-irritable locations in a tight muscle band, usually appearing as palpable nodules and can occur for different conditions, and the central hypothesis is called the integrated hypothesis [1, 2, 5]. The integrated hypothesis occurs through a vicious cycle initiated by repetitive microtraumas, tensions, acute traumas, excessive contractions, and wrong posture, which can cause injuries to the sarcoplasmic reticulum, alteration of calcium, acetylcholine, and adenosine triphosphate concentrations, alteration in muscle filaments, as well as depolarization and muscle contraction, which will result in the inflammatory process and decrease in local circulation [2, 5].

Individuals with MTrPs may have sensory, motor, and autonomic changes, such as hypersensitivity to pain, increased muscle contraction, change in range of motion (ROM), muscle weakness, and changes in peripheral circulation and balance [4, 7, 8]. The most common symptoms are pain and local muscle contraction, which may appear simultaneously [8].

Pain can manifest as spontaneously and continuously, which characterizes an active MTrPs, or it can appear only during compression of the region, characterizing the latent MTrPs. Also, can be localized or referred—to when it propagates along the nervous path of the muscles affected by the MTrPs -, in both types of MTrPs [1, 2, 7, 8].

The diagnosis of MTrPs is based on evaluation and palpation of the muscles, having as criteria the presence of at least three of the following conditions: (A) Tight muscle band, (B) Hypersensitive point, (C) Local or referred pain, and (D) Local muscle contraction response [5, 8, 9]. In addition, infrared thermography can also be used, which is a non-invasive imaging technique that allows the capture of infrared radiation emitted by the body, allowing the reproduction of the image of the designed region in real-time with different temperature gradients [10–12].

The literature presents several treatments for trigger points, which may or may not be invasive [5, 13]. Some of the non-invasive treatments are ischemic compressions (IC), electrostimulation, laser therapy, and extracorporeal shockwave therapy (ESWT) [5, 13–15]. The literature has shown that IC and dry needling are the most used treatment techniques for the treatment of MTrPs and were superior to placebo [16]. IC consists of pressure applied to the MTrPs until the discomfort reduces and the tense muscle relaxes [2, 13, 17, 18]. IC results in sarcomere lengthening, analgesia, decreased tension, and increased ROM [16, 19–21].

ESWT consists of the emission of a high-energy sound wave released by equipment [22, 23]. This wave propagates through the epithelial tissue, causing microlesions, micro ruptures of capillaries, and an increase in chemical mediators through the cavitation process that results in decreased pain and inflammation, breaking the vicious cycle of contraction and tissue regeneration [15, 22–25].

The IC and ESWT have been shown to be efficient in increasing the pain threshold and decreasing pain intensity [14–16], however, the studies that compare these techniques are mostly performed in the trapezius muscle, in the upper fibers [26, 27], and limited to the muscles of the lower limbs, which are of great importance and are commonly injured [2, 4, 18]. Therefore, the study aims to compare the effects of IC and ESWT on pressure pain threshold immediately after the intervention and after 48h.

## Materials and methods

### Design

**Fig 1:**
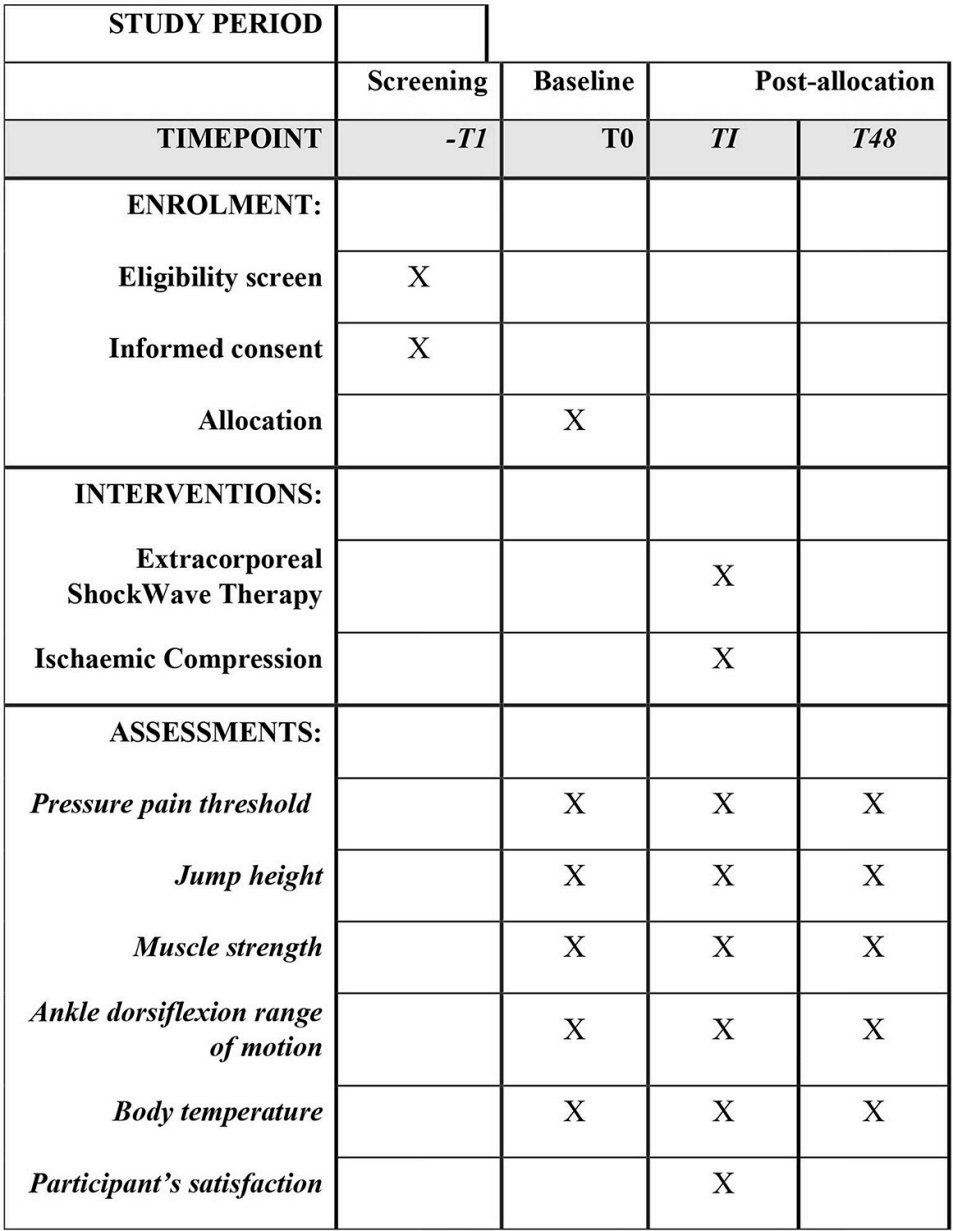
Standard protocol items: Recommendations for International Trials statement SPIRIT.

A two-arm, parallel randomized, double-blinded (outcome assessor and statistician), will be performed. The study follows the TIDieR (Template for Intervention Description and Replication checklist) [28], and the 2013 Standard Protocol Items: Recommendations for International Trials statement [29] (Fig 1).

### Ethical aspects

The study was approved by the Research Ethics Committee of the Faculty of Philosophy and Sciences, São Paulo State University, the campus of Marília (CAAE 46682921.9.0000.5406) and will comply with the resolution 466/2012 of the National Health Council. The randomized controlled trial will follow the CONSORT recommendations and is registered in the Brazilian Clinical Trials Registry (RBR-6wryhb9). Participants will be informed about all study procedures and asked to sign the Informed Consent Form prior to their enrollment in the study.

### Participants

#### Recruitment

Recruitment of participants will be carried out through social media, partnerships between the public health service and the university, and sports clubs that will publish and share the trial.

### Eligibility criteria

#### Inclusion and exclusion criteria

*Eligibility criteria*. Age between 18 and 45 years old, both genders, presence of MTrPs in the triceps surae muscle, be “active” or “very active” according to the International Physical Activity Questionnaire (IPAQ), and have signed the informed consent form.

*Non-eligibility criteria*. Body Mass Index (BMI) > 30, having taken analgesic, anti-inflammatory, anticoagulant, muscle relaxant, or antipyretic medication up to 24 hours before the study, having ingested stimulants, such as caffeine and alcohol up to 8 hours before the study, have practiced strenuous physical exercises up to 24 hours before the study, and mental and sensitivity changes that may be harmful to the participant or may cause alterations in the results.

### Procedures

#### Baseline evaluation (T0)

Participants will be evaluated before the treatment (T0), anamnesis will be carried out with the collection of personal data (age, sex, BMI, height, previous history of injuries, diseases, and use of continuous medication); which physical exercises the participants practice and the regularity. Pressure pain threshold will be evaluated by a pressure algometer (Digital Manual Dynamometer, model DDK/20—Kratos), that contains a bar with a flat circular tip of 1.0cm^2^ in diameter, with digital reading and accuracy of 0.005 Kg. The circular tip will be used to apply pressure on the MTrPs until the pain is reported. The value will be fixed on the equipment screen and noted. Three tests will be performed at each point and the average will be calculated [30].

After evaluating the pressure pain threshold, the lower limbs will be warmed up on the treadmill, with a 6-minute walk protocol, monitoring the participants’ heart rate, between 55% and 60% of the maximum heart rate, using a digital heart monitor. The maximum heart rate will be found by the Karvonen calculation (HRmax = 220 –age) [31].

For the assessment using infrared thermography, participants will be instructed to stand up straight and in a comfortable position for 10 minutes in a room with a controlled temperature (22°C±1°C) to perform the first infrared thermography [32, 33]. The FLIR E8 WI-FI thermographic camera (FLIR® Systems, Inc.), with a resolution of 320x240 pixels, with sensors that allow the evaluation of temperatures in the ranges of -20°C to +250°C, with a sensitivity of <0.06°C and accuracy of ±2°C, will be used. Two photos will be taken in each position, with one meter of the demarcated area, at angles of 45°, 0°, and -45°.

Subsequently, palpation of the triceps surae will be performed by two trained evaluators to verify the presence of MTrPs. When the presence of the MTrPs is confirmed, the two most painful points will be marked with a permanent pen. The second thermography will be carried out. Two photos will be taken in each position, at angles of 45°, 0°, and -45°, in minutes 5 and 10. During the 10 minutes of waiting for thermography, participants will be instructed to answer the IPAQ, which allows the evaluator to check and calculate the weekly time that participants practice light, moderate or vigorous exercise, whether for leisure, work, or transportation [34, 35].

The Weight-Bearing Lunge Test (WBLT) will be used to assess the ankle dorsiflexion ROM. Participants will be positioned standing in front of the wall, with the limb being evaluated on a measuring tape, next to the wall, and will be asked to try to touch the knee on the wall keeping the heel on the floor, if this is possible, participants will be instructed to place the foot away from the wall [36, 37]. When the maximum position is found, a smartphone with the Clinometer® software will be positioned in the middle of the tibial and will be instructed to perform three movements to calculate the average angle [36, 38].

The vertical jump analysis will be performed through the counter movement jump (CMJ), which consists of a quick knee flexion followed by a knee extension with the hands-on hip [39]. Participants will be positioned on a contact platform (Elite Jump System®; S2 Sports, São Paulo, Brazil), and will be instructed on how to perform the jump and will have three attempts for familiarization with 15 seconds of rest between them [39]. Subsequently, will be requested more three attempts that will be considered valid jumps, with 15 seconds of rest.

Subsequently, the muscle strength will be evaluated by Biodex® isokinetic dynamometer, System 4 model (Biodex Medical Systems, New York–USA). Familiarization will be performed with five repetitions. The test will be performed bilaterally in the concentric/concentric mode of plantar flexion and dorsiflexion, at speeds of 60°/s and 90°/s, with five repetitions at each of the speeds and with forty-five seconds of rest between them [40, 41]. Participants will be positioned, according to the manual of Biodex, seated, with 75° of hip flexion, and 30° of knee flexion, and will be stabilized on the trunk and thigh of the contralateral lower limb, to avoid compensation. The evaluated lower limb will be positioned to align the axis of the dynamometer with the lateral malleolus and then the measurement of the maximum ROM in plantar flexion and dorsiflexion will be performed, and finally, the limb will be weighed [42].

#### Immediate evaluation after treatment (T1)

T1 will be carried out right after the treatment and the evaluation described in T0 will be redone, except the 6-minute walk protocol on treadmill. The participants will answer the MedRisk questionnaire, which evaluates the participant’s satisfaction and is considered a marker of the quality of service provided [43]. The questionnaire consists of 13 questions that aim to qualify the service and the environment in which the service was provided, with a score from 0 to 5 or not applicable [43].

#### Evaluation after 48 hours (T48)

It will take place 48 hours after treatment and, as in T1, the assessment will be redone, including the 6-minute walk protocol on treadmill.

### Research team

The trial will involve five researchers: three researchers responsible for evaluations. One researcher will be responsible for randomizing participants before the recruitment and applying the treatment. One researcher will perform the statistical analysis.

### Sample size and power analysis

The sample size was calculated using the G*Power 3.1.9.7 software (G*Power©, Dusseldorf University, Dusseldorf, Germany). Due to the novelty of the proposed treatment and the lack of data in the literature, we carried out a pilot study. Algometry data (pressure pain threshold) of MTrPs found in the triceps surae muscle obtained from 48 hours after treatment (n = 12), were used to estimate sample size. Considering Student’s t-test, differences between two independent means (two groups) and the pilot test, the results of both groups were expressed as means and standard deviations. It was used the algometry in the ESWT group (2.45±0.79 kg/cm^2^) and IC group (3.84±1.56 kg/cm^2^), an effect size of 1.12, power of 0.95, error probability α 0.05, with an allocation ratio of N2/N1 of 1 and a sample loss of 10%. Considering the above-mentioned sample size analysis, 40 participants, 20 in each group, will be necessary.

### Randomization, allocation, and blinding

**Fig 2:**
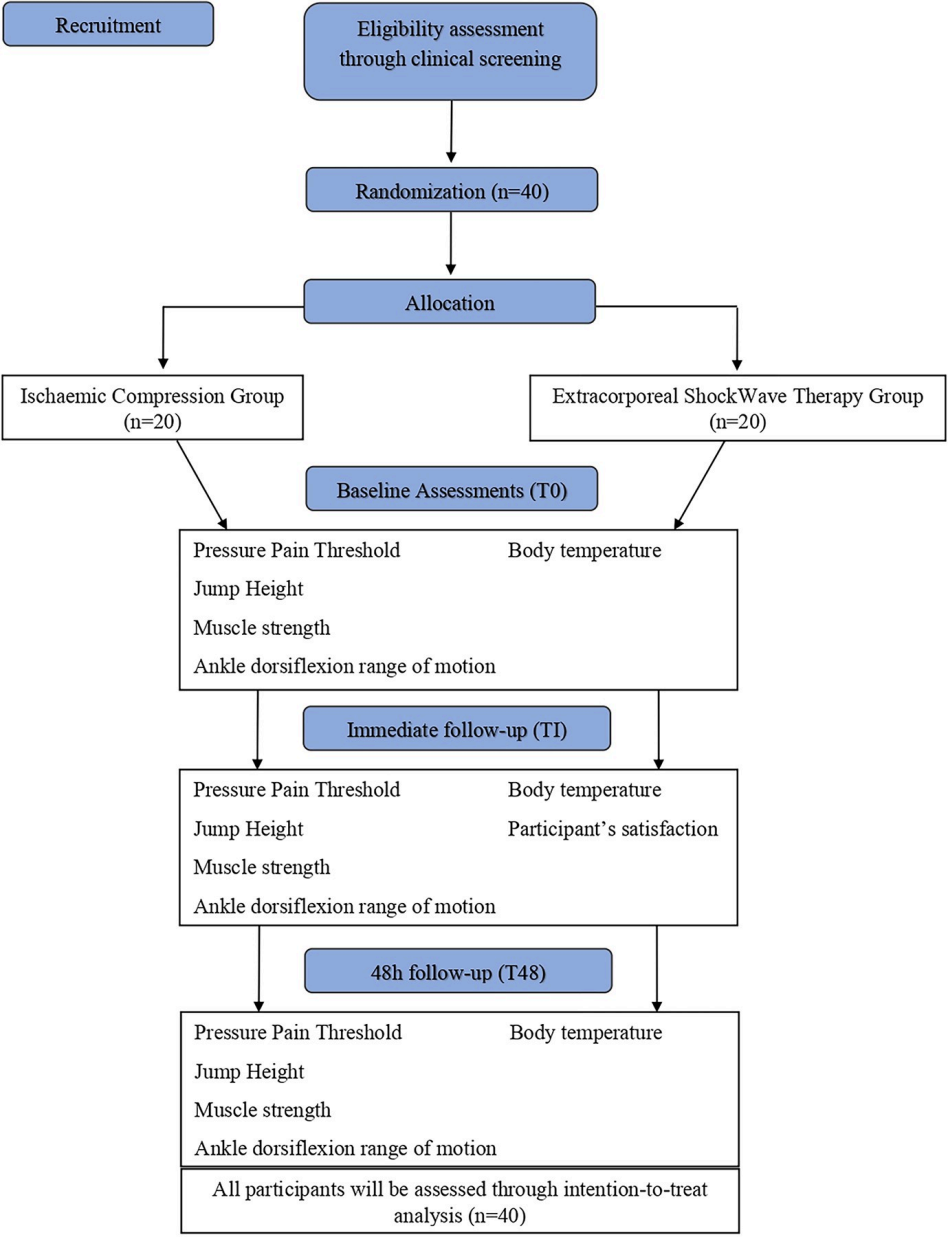
Study fluxogram.

Randomization will be done on the randomization plans website, in in 5 blocks of 8 participants, before recruitment. Fig 2 shows how the participants will be randomly allocated into two groups: (A) ESWT Group (ESWTG; n = 20); (B) IC group (ICG; n = 20) and the evaluators will not have access to this information. Participants will follow the therapist responsible for the intervention to a separate room and will be instructed not to make any comments about the group that was assigned, thus ensuring the blinding of the evaluators.

### Outcomes measures

#### Primary outcomes

The primary outcome is the pressure pain threshold that will be assessed at T0, T1, and T48. The evaluation will be carried out through the pressure algometer. The equipment will be used to apply pressure on the demarcated points and the value will be marked as soon as the participants report that the stimulus has become painful [30].

#### Secondary outcomes

There will be five secondary outcome measures: 1) Jump height, 2) Muscle strength, 3) Ankle dorsiflexion range of motion, 4) Body temperature and 5) Participant’s satisfaction.

1. The jump height will be evaluated through the jump analysis performed on the Elite Jump System^®^ jumping platform. It will be performed at T0, T1, and T48.
2. Muscle strength will be evaluated using a Biodex^®^ isokinetic dynamometer, model System 4, in concentric/concentric mode at two speeds, allowing the evaluation of plantar flexion and dorsiflexion movements, performed mainly by the tibialis anterior muscle and triceps surae muscle, respectively. It will be performed at T0, T1, and T48.
3. The dorsiflexion ROM will be evaluated using the WBLT, which will be performed at T0, TI, and T48. Participants will be asked to stand on top of a measuring tape, with continuous numbering in centimeters, in front of the wall, and perform the movement of touching the knee to the wall without removing the heel from the floor and/or rotating the pelvis. At the same time, a smartphone with the Clinometer® app will be positioned on the tibia, thus obtaining the maximum angulation of dorsiflexion [36].
4. The correlation of MTrPs with temperature will be performed at T0, T1, and T48 and through the images obtained by thermography before palpation and after palpation, allowing to assess whether points with higher or lower temperatures correspond or not to MTrPs found during palpation performed by the evaluators.
5. The participants will answer the MedRisk questionnaire, which evaluates the participant’s satisfaction and is considered a marker of the quality of service provided [43]. The questionnaire consists of 13 questions that aim to qualify the service and the environment in which the service was provided, with a score from 0 to 5 or not applicable [43]. It will be performed only at T1.

### Interventions

#### Extracorporeal shockwave therapy group

Participants will undergo ESWT, performed by the Thork Shock Wave® device from IBRAMED. The parameters will be 2000 shots at 10Hz and an energy flux density of 60 mJ/mm2. The application will be carried out with the head perpendicular, 2.5 mm metal tip, using gel on each of the demarcated points with slow circular movements. The intervention will be individual, performed only once at each point, lasting 3 minutes per point. Participants will be instructed to inform the therapist if there is pain or discomfort, and if so, the procedure will be interrupted.

#### Ischemic compression group

Participants will be subjected to digital pressure with gradual and increasing force on the demarcated MTrPs. Compression will be maintained for 90 seconds, performed only once at each point, and will be individual. A trained therapist will be in constant communication with the participants to verify the presence of discomfort or pain, from the moment the pressure applied by the therapist becomes pain it will be maintained, and as the sensation of pain and muscle tension decreases, the pressure applied will increase.

### Statistical analysis

It will be a superiority trial. Statistical analysis will follow the concepts of intention-to-treat analysis. Statistical analysis will be performed using the commercial software Biostat® 2009 Professional 5.8.4 for Windows and will be performed by a statistician not involved in the study. The Chi-square test or Fisher’s exact test will be applied to compare the nominal data between groups. For data analysis, after confirming the normality of data distribution and homogeneity of variances, the appropriate statistical tests will be selected. Primary and secondary outcomes will be tested using a 2-way general linear model (GLM), with Group (ESWT and IC group) and Time Point (T0, T1 and T48) as factors, with repeated measures on the time point factor (i.e. within-subject). The interaction effect of group by time point will be the primary test of interest. The hypothesis of sphericity will be tested by the Mauchly test and when the sphericity should be rejected the Greenhouse-Geisser correction will be applied. When a significant main effect or interaction effect will be found, pair-wise post-hoc tests will be applied using Bonferroni correction and relative percentages will be used to show the magnitude of differences on the statistical tests. If any baseline characteristic showed differences between groups they will be included as covariates in univariate GLM against outcome variables. Univariate linear regressions will be conducted between each of the independent variables and algometry (pressure pain threshold) of MTrPs. The variables detected as significant in univariate linear regressions will be included in multivariate liner regressions. Differences in proportions in satisfaction score measured by Medrisk questionnaire will be presented in mean and standard deviation and compared between groups by independent t-test. Spearman’s correlation coefficient will be used to determine the interaction between MTrPs with temperature. Differences will be considered statistically significant if p<.05.

## Discussion

Musculoskeletal pain is the main reason for seeking health care, and one of the most common reasons is called myofascial pain resulting from the emergence of MTrPs [3, 17]. Treatments by IC or ESWT are found in the literature for the treatment of MTrPs [27]. According to Gemmell, Miller, and Nordstrom (2008), IC was more effective than placebo for pain reduction in patients with trapezius muscle MTrPs [44], this was also proven by Kisilewicz et al. (2018), adding that a single session with IC is able to decrease muscle tension and consequently increase ROM [21].

Regarding ESWT, Király, Bender, and Hodosi (2018) concluded that, like a low-frequency laser, ESWT promotes increased pain tolerance and improved function in the cervical spine in patients with MTrPs in the trapezius muscle, upper fibers [23]. Zhang et al (2020) performed a systematic review with meta-analysis and found similar results [25].

Mushtaq, Pattnaik, and Mohanty (2017) compared ESWT and IC on MTrPs found in the upper trapezius muscle, and resulted in decreased pain intensity, increased pressure pain threshold, and increased ROM of cervical flexion, with better performance in the group treated with ESWT [27].

Despite published studies, there is no consensus on the effect of ESWT and IC on the triceps surae muscle. Thus, the study hypothesizes that there will be an increase in the pressure pain threshold after the application of treatments, however, the ESWT group should be better due to findings in the literature about the effects of nitric oxide regulation, responsible for the improvement in local circulation and consequently in nutrition and inflammation, and also stimulates tissue and vascular regeneration [23, 25, 45].

## Trial status

At the time of study submission, participants were being recruited.

## Supporting information

S1 Checklist SPIRIT 2013 checklist: Recommended items to address in a clinical trial protocol and related documents. (DOC)

S1 File (PDF)

S2 File (PDF)
