# Supplementary material for: Effect of ischemic compressions versus extracorporeal shockwave therapy on myofascial trigger points: A protocol of a randomized controlled trial
Source: PLoS One. 2023 Mar 30;18(3):e0283337. doi: 10.1371/journal.pone.0283337 (PMC10062556; doi:10.1371/journal.pone.0283337)
Supplement: S1 File — (PDF) [file pone.0283337.s002.pdf]

OPINION CONSTITUTED FROM THE CEP

RESEARCH PROJECT DATA

Search Title: EVALUATION OF DIFFERENT METHODS USED IN TREATMENTS IN MYOFASCIAL TRIGGER POINTS: RANDOMIZED CONTROLLED ASSAY

Researcher: CRISTIANE RODRIGUES PEDRONI

Thematic Area:

Version: 1

CAAE: 46682921.9.0000.5406

Proposing Institution: Faculty of Philosophy and Sciences / UNESP - Campus of Marília

Main Sponsor: Own Funding

OPINION DATA

Opinion Number: 4,761,425

Project Presentation:

Currently, one of the main complaints of pain made by the population comes from the musculoskeletal system, one third corresponding to Myofascial Pain Syndrome (SDM). Patients with MDS are the most frequent users of the primary sector of the country's unified health system, taking into account that MDS can affect any individual, regardless of ethnicity, gender and age, and its appearance is more common in young people and adults, between 27 and 27. 50 years. (BARBERO et al., 2019; RICKARDS, 2006; SHAH et al., 2015; TANTANATIP; CHANG, 2020). The SDM has as its main characteristic the appearance of trigger points (PG) in the muscular region (SIMONS et al., 1999) . PG is commonly defined as a hyperirritable location in a tight muscle band. (FERNÁNDEZ-DE-LAS-PENAS; DOMMERHOLT, 2018; ARENDT-NIELSEN, 2011; PARK et al., 2010; SIMONS et al., 1999). Patients with PG may present as symptoms sensory changes, such as hypersensitivity to pain; motor, such as decreased range of motion (ROM), altered coordination, muscle weakness, decreased joint stability and function; and autonomic symptoms, such as changes in peripheral circulation, balance disorders, and vomiting. (FERNÁNDEZ-DE-LASPENAS; DOMMERHOLT, 2018; PARK et al., 2010; SANTOS et al., 2012). Among the symptoms mentioned, the most common are contraction of the local muscles and pain, which may appear simultaneously . The presentation of pain can occur spontaneously and continuously, characterizing an active TP or only when compression is applied to the region, characterizing latent TP. In addition, the pain can be local,

that is, concentrated only in the region where the PG is found or referred, when it propagates, normally occurring in the nervous path of the innervation of the affected muscle. (SIMONS et al., 1999; GERWIN, 2014; PARK et al., 2010). PG can be classified according to its state divided into active or acute and latent or chronic (NIEL-ASHER, 2008). Regarding its location, the PG can be classified as central or primary, which are located in the muscle belly and considered the first to form. Later it is common the appearance of the called PG satellites or secondary next to the primary ones. There are fixation devices that are located in the musculature closer to the tendon than in the muscle belly. Finally, there are diffuse PGs, which occur mainly when there are high deformities, originating in a diffuse way in large quantities in the lines of deformity and overload (NIEL-ASHER, 2008). The pathophysiology of PG has not yet been established and there are different hypotheses about its origin. The main hypothesis was named as energy crisis of muscle fibers, which reports that the development of PG starts from an acute trauma or repetitive microtrauma, which will cause an overload of muscle fibers leading to changes in the musculature, local circulation and permeability. of the cell membrane and as a result there is the appearance of an inflammatory process, decreased local blood circulation, increased metabolic needs, changes in muscle contraction and shortening of sarcomeres (ALVAREZ; ROCKWELL, 2002; NIEL-ASHER, 2008; SHAH et al. ., 2015; BRON; DOMMERHOLT, 2012). Possible microtraumas that can cause the appearance of PG are surgicalscars under tension, poor posture, sedentary lifestyle, muscle overload, excessive stretching or shortening, lack of vitamins, such as, for example, vitamin C and D, sleep changes and stress. In addition, very fast movements and sudden traumas, such as direct trauma, falls, accidents can also cause the appearance of PG due to the high energetic load of contraction that occurs (ALVAREZ; ROCKWELL, 2002; SANTOS et al., 2012). There is no consensus on the evaluation and diagnosis criteria. for the PG, it is known that they should be based on a goodassessment. The current gold standard for diagnosis is palpation performed during physical examination, whichis considered the most accessible method and can be performed by compressing a band.

tense muscle or a muscle knot with the digital pulp or by forceps. However, this method is not always the most reliable and can be subjective, taking into account that it requires professional experience in identifying PG, associated with characteristic signs and symptoms. On the other hand, imaging tests such as ultrasound (US) and thermography, when performed by trained professionals, are considered more objective and can easily identify even small-sized PG that would be difficult to locate through palpation (FERNÁNDEZ-DE-LASPENAS; DOMMERHOLT, 2018; GERWIN, 2014; KUMBHARE et al., 2017).

US is a non-invasive diagnostic technique that provides real-time imaging of the region that is being analyzed. This image shows the muscle, tendon, fascia, fat and other soft tissues that make up the site. The most used mode for viewing PG is called B Mode. PG can vary in size and are usually located in muscle tissue, its shape is usually elliptical and appears in a discrete, focal and hypoechoic manner. There are studies that compare, in addition to the degree of echogenicity, hemodynamic changes and tissue texture, allowing the classification and presence of PG (KUMBHARE et al., 2017; SIKDAR et al., 2010; TURO et al., 2013). Infrared radiation (IRT), like ultrasonography, is a non-invasive method that allows the reproduction of an image of the region, in real time and in an objective way, with the different gradients of radiated heat temperature,from the recording of infrared radiation. . Infrared radiation is heat energy constantly released by any body or object as a result of the movement and agitation of its molecules. In addition, IRT does not emit radiation and there is no need for the therapist to contact the patient, making it safer (BRIOSCHI et al., 2003; GABRIEL et al., 2016; MARINS et al., 2015). However, it has its limitations, such as factors that influence temperature, from environmental factors such as air conditioning and humidity; individual factors, such as fat percentage, sex, among others. Sites that present PG tend to appear in the IRT in warmer tones, due to an inflammatory process causing an increase in local temperature (BANDEIRA et al., 2012; HOLEY; DIXON; SELFE, 2011; MERLA et al., 2010; PRIEGO QUESADA et al. al., 2015; MARINS et al., 2015). Consideration should be given to the treatment of PG, its formation and classification, emotional factors such as stress and muscular and postural changes. The treatment is diverse and can be performed by one or more associated techniques. The techniques can be invasive, such as dry needling and or with associated medications, or non-invasive, such as postural education and body awareness; manual therapy, such as ischemic compression, exercises, stretching, among others; and electrophototherapy, which includes shock waves, therapeutic ultrasound, laser therapy, among others (BORG- STEIN; IACCARINO, 2014; GALASSO et al., 2020; RICKARDS, 2006). Known as Dry Needling, it consists of perforating the skin, superficial and muscular tissues using a fine needle. With the application of the needle, thereis a microlesion that will activate three mechanisms, the neurophysiological, the chemical and the mechanical, which will result in a reduction of hypersensitivity to pain, the elongation of the sarcomeres, local chemical homeostasis and improvement of the circulation close to the PG. In addition, it occurs

increased pressure pain threshold and improved muscle function. (GATTIE; CLELAND; SNODGRASS, 2017; ZIAEIFAR et al., 2013; CAGNIE et al., 2012; DOMMERHOLT, 2004). results in a decrease in algogenic substances, present in the formation of PG, in addition to a reduction in the activation of nociceptors. Adverse effects that can occur with the application of the needle are reproduction and/or momentary increase in pain, perforation of small vessels with eventual bleeding, formation of small ecchymoses and local hyperemia reaction (CARVALHO et al.2017). Ischemic compression (IC) is a treatment used to release the PG, it consists of applying pressure under the PG until the discomfort subsides. More pressure can be increased until the complete release of the PG. It causes elongation of the sarcomeres, improves blood flow by removing harmful chemicals which results in reduced local muscle tension, increased ROM, pain relief and increased pressure pain threshold (GRIEVE et al., 2013; RICKARDS, 2006). ; SIMONS, 2004; TABATABAIEE et al., 2019). Shockwave Therapy (OCD) is performedby equipment that emits a high-energy sound wave produced by the release of high pressure air. This mechanical energy propagates in the tissue and causes micro-lesions, micro-ruptures of capillaries and chemical mediators through the cavitation mechanism, with this, the result is an improvement in revascularization and tissue regeneration, reduction of inflammation, analgesia, in addition to the release of PG, taking into account that theacoustic wave unlocks the calcium pump, decreasing the constant contraction cycle (KIRÁLY; BENDER; HODOSI,2018; KISCH et al., 2016; PARK et al., 2018; TAHRIRIAN et al., 2012; WANG; WANG;

YANG, 2004). AS, CI and OCD therapies are some of the therapies that are found in the literature, however there are still gaps regarding the best resources and parameters for the treatment of myofascial trigger points. Thus, the aim of this study will be to compare the effects of a treatment session of myofascial trigger points with AS, CI and OCD after 1 session and after 48 hours on pain levels, body temperature, imaging and strength.

Research Objective:

Primary Objective: To

compare the acute and chronic effects of three techniques in the treatment of myofascial trigger points.

Secondary Objective:
To analyze the effects of ischemic compression on PG; To
analyze the effects of shockwave therapy on PG; Analyze the
effects of dry needling on PG; To verify the effect of the techniques
on the variables of pain, strength, range of motion, electrical activity and performance (function) of the evaluated segment.
Check the agreement between thermography and ultrasound images with digital palpation of trigger points.

Check the relationship of therapies with muscle strength and PG pain;

Assessment of Risks and Benefits:

Scratchs:

Because it is a muscle condition, whose treatment is based on microlesions of the trigger point or tension nodule, it is possible that muscle pain occurs at the time of application of the technique and a few hours later. However, despite the anticipated discomfort, none of the interventions generate known risks of relevant damage to muscle function or structure.

Benefits:

The direct benefits provided to the research subject concern the improvement of muscle function after the application of treatment techniques,
considering that all are proven effective, causing analgesia, improvement of local circulation and muscle function. The indirect benefits relate to greater knowledge about the techniques used to treat myofascial trigger points, helping to increase scientific knowledge applied to clinical practice.

Comments and Considerations on the Research:

This is research with a relevant theme, with a well-structured methodological design.

Considerations on the Mandatory Submission Terms:

Appropriate mandatory terms.

Recommendations:

There are no recommendations.

Conclusions or Pending and List of Inadequacies:

Considering the documentation presented, the project can be considered approved.

Final Considerations at the discretion of the CEP:

The CEP of the FFC of UNESP de MARÍLIA, in an ordinary meeting of 05/19/2021, after accepting the opinion

of the reporting member previously approved for the present study and in compliance with all provisions of resolutions 466/2012, 510/2016 and complementary ones, as well as having approved the Consent Form
Free and Clear, as well as all attachments included in the survey, resolves to APPROVE the survey "EVALUATION OF DIFFERENT METHODS USED IN TREATMENT IN TRIGGER POINTS MYOFASCIAL: RANDOMIZED CONTROLLED ASSAY".

This opinion was prepared based on the documents listed below:

Document Type File	post	Author	Situation
Basic Information of the Project PB_INFORMAÇÕES_BÁSICAS_DO_P ROJETO_1751318.pdf Declaration of AuthorizationPesquisa.pdf Institution and Infrastructure / Investigator Brochure TCLE / Detailed Terms of Assent / Justification of Absence Cover Sheet	05/10/2021 20:58:42<br>05/10/2021 20:52:29	CRISTIANE RODRIGUES PEDRONI	Accepted<br>Accepted
Project.pdf	05/10/2021 20:51:58	CRISTIANE RODRIGUES PEDRONI	Accepted
ICF.pdf	05/10/2021 20:49:36	CRISTIANE RODRIGUES PEDRONI	Accepted
FolhaRosto.pdf	05/10/2021 20:48:52	CRISTIANE RODRIGUES PEDRONI	Accepted

Opinion Status:
Approved

Requires CONEP Appraisal:
No

MARILIA, June 9, 2021

Signed by:
SIMONE APARECIDA CAPELLINI
(Coordinator)

Address: Av. Hygino Muzzi Filho, 737
Neighborhood: University Campus **ZIP CODE:** 17,525-900
State: SP **Municipality:** MARILIA
Telephone: (14)3402-1346 **Email:** cep.marilia@unesp.br
