# Supplementary material for: Effect of ischemic compressions versus extracorporeal shockwave therapy on myofascial trigger points: A protocol of a randomized controlled trial
Source: PLoS One. 2023 Mar 30;18(3):e0283337. doi: 10.1371/journal.pone.0283337 (PMC10062556; doi:10.1371/journal.pone.0283337)
Supplement: S2 File — (PDF) [file pone.0283337.s003.pdf]

PARECER CONSUBSTANCIADO DO CEP

DADOS DO PROJETO DE PESQUISA

Título da Pesquisa: AVALIAÇÃO DE DIFERENTES MÉTODOS UTILIZADOS NO TRATAMENTOS NOS PONTOS GATILHOS MIOFASCIAIS: ENSAIO CONTROLADO ALEATORIZADO

Pesquisador: CRISTIANE RODRIGUES PEDRONI

Área Temática:

Versão: 1

CAAE: 46682921.9.0000.5406

Instituição Proponente: Faculdade de Filosofia e Ciências/ UNESP - Campus de Marília

Patrocinador Principal: Financiamento Próprio

DADOS DO PARECER

Número do Parecer: 4.761.425

Apresentação do Projeto:

Atualmente, uma das principais reclamações de dor feita pela população é advinda do sistema musculoesquelético, sendo um terço correspondente à Síndrome da Dor Miofascial (SDM). Pacientes com SDM são os maiores frequentadores do setor primário do sistema único de saúde do país,levando em conta que a SDM pode acometer qualquer indivíduo, independente da etnia, gênero e idade, sendo mais comum seu aparecimento em jovens e adultos, entre 27 e 50 anos. (BARBERO et al., 2019; RICKARDS, 2006; SHAH et al., 2015; TANTANATIP; CHANG, 2020). A SDM tem como sua principal característica o aparecimento de pontos-gatilho (PG) na região muscular (SIMONS et al., 1999) . O PG é definido, comumente,como um local hiper irritável em uma banda muscular tensa. (FERNÁNDEZ-DE-LAS-PENAS; DOMMERHOLT, 2018; ARENDT-NIELSEN, 2011;PARK et al., 2010; SIMONS et al., 1999). Pacientes com
PG podem apresentar como sintomas alterações sensoriais, como hipersensibilidade a dor; motoras, como diminuição da amplitude de movimento (ADM), alteração da coordenação, fraqueza muscular, diminuição da estabilidade articular e da função; e sintomas autonômicos, tais como alterações da circulação periférica, alterações de equilíbrio e vômitos. (FERNÁNDEZ-DE-LASPENAS; DOMMERHOLT, 2018; PARK et al., 2010; SANTOS et al., 2012).Dentre os sintomas mencionados, os mais comuns são a contração da musculatura local e a dor, que podem aparecer simultaneamente . A apresentação da dor pode ocorrer de maneira espontânea e contínua, caracterizando um PG ativo ou somente no momento em que é aplicado uma compressão da região, caracterizando o PG latente. Além disso, a dor pode ser local,

ou seja, concentrada apenas na região onde se encontra o PG ou referida, quando esta se propaga, normalmente ocorrendo no trajeto nervoso da inervação do músculo acometido. (SIMONS et al., 1999; GERWIN, 2014; PARK et al., 2010).O PG pode ser classificado quanto ao seu estado dividido em ativo ou agudo e latente ou crônico (NIEL-ASHER, 2008). Já com relação a sua localização, o PG pode ser classificado

como central ou primário, que são localizados no ventre muscular e considerados os primeiros a se formar. Posteriormente é comum o aparecimento dos PG chamados satélites ou secundários próximos aos primários. Existem os de fixação que são localizados na musculatura mais próxima ao tendão do que no ventre muscular. Por fim, existem os PG difusos, que ocorrem principalmente quando se tem altas deformidades, se originando de maneira difusa em grande quantidade nas linhas de deformidade e de sobrecarga (NIEL-ASHER, 2008).A fisiopatologia do PG ainda não foi estabelecida e há diferentes hipóteses sobre sua origem. A principal hipótese foi nomeada como crise energética das fibras musculares, que relata que o desenvolvimento dos PG inicia a partir de um trauma agudo ou microtraumas repetitivos, que vai ocasionar uma sobrecarga das fibras musculares levando a alterações na musculatura, na circulação local e na permeabilidade da membrana celular e como resultado há o aparecimento

de um processo inflamatório, diminuição da circulação sanguínea local, aumento das necessidades metabólicas, alterações na contração da musculatura e encurtamento dos sarcômeros (ALVAREZ; ROCKWELL, 2002; NIEL-ASHER, 2008; SHAH et al., 2015; BRON; DOMMERHOLT, 2012).Os possíveis microtraumas que podem ocasionar o aparecimento do PG são cicatrizes cirúrgicas sob tensão, má postura, sedentarismo, sobrecarga muscular, excesso de alongamento ou encurtamento, falta de vitaminas, como por exemplo, a vitamina C e D, alterações de sono e estresse. Além disso, movimentos muito rápidos e bruscos, como traumas diretos, quedas, acidentes também podem causar o aparecimento de PG pela alta carga enérgica de contração que ocorre (ALVAREZ; ROCKWELL, 2002; SANTOS et al., 2012).Não há consenso sobre os critérios de avaliação e de diagnóstico para o PG, sabe-se que devem ser baseados em uma boa avaliação. O atual padrão ouro para realizar o diagnóstico é a palpação realizada durante o exame físico, considerado o método mais acessível e que pode ser feito por meio da compressão de uma banda muscular tensa ou de um nó muscular com a polpa digital ou pela pinça. Entretanto, esse método nem sempre é o mais confiável e pode ser subjetivo, levando em conta que exige experiência do profissional na identificação do PG, associado aos sinais e sintomas característicos. Já os exames de imagem, como a ultrassonografia (US) e a termografia, quando realizados por profissionais treinados, são considerados mais objetivos e podem identificar com facilidade até PG de pequenos tamanhos que

Endereço: Av. Hygino Muzzi Filho, 737
Bairro: Campus Universitário **CEP:** 17.525-900
UF: SP **Município:** MARILIA
Telefone: (14)3402-1346 **E-mail:** cep.marilia@unesp.br

seriam de difícil localização por meio da palpação (FERNÁNDEZ-DE-LASPENAS; DOMMERHOLT, 2018; GERWIN, 2014; KUMBHARE et al., 2017).A US é uma técnica não invasiva de diagnóstico que fornece imagem em tempo real da região que está sendo analisada. Nessa imagem é mostrado a parte muscular, tendínea, a fáscia, gordura e outros tecidos moles que fazem a composição do local. O modo mais utilizado para a visualização dos PG é chamado de B Mode. O PG pode variar de tamanho e normalmente são localizados no tecido muscular, seu formato costuma ser elíptico e aparece de maneira discreta, focal e hipoecóica. Há estudos que comparam além do grau de ecogenicidade, as alterações hemodinâmicas e de textura do tecido, permitindo a classificação e presença do PG (KUMBHARE et al., 2017; SIKDAR et al., 2010; TURO et al., 2013).A infravermelha (IRT), assim como a ultrassonografia, é uma método não invasivo que permite a reprodução de uma imagem da região, em tempo real e de maneira objetiva, com os diferentes gradientes de temperatura irradiada de calor, a partir do registro da radiação infravermelha. A radiação infravermelha é a energia térmica liberada constantemente por qualquer corpo ou objeto, como resultado do movimento e agitação de suas moléculas. Além disso, a IRT não possui emissão de radiação e não é necessário contato do terapeuta com o paciente, tornando-o mais seguro (BRIOSCHI et al., 2003; GABRIEL et al., 2016; MARINS et al., 2015). Entretanto, ela tem suas limitações, como os fatores que influenciam na temperatura, desde fatores ambientais, tais como ar condicionado e umidade; a fatores individuais, como porcentagem de gordura, sexo, entre outros. Locais que apresentam PG costumam aparecer na IRT em tons mais quentes, decorrente de um processo inflamatório ocasionando o aumento da temperatura local (BANDEIRA et al., 2012; HOLEY; DIXON; SELFE, 2011; MERLA et al., 2010; PRIEGO QUESADA et al., 2015; MARINS et al., 2015).Deve-se levar em consideração para o tratamento do PG, sua formação e classificação, fatores emocionais, como o estresse e alterações musculares e posturais. O tratamento é diversificado e pode ser realizado por uma ou mais técnicas associadas. As técnicas podem ser invasivas, como o agulhamento a seco e ou com medicamentos associados ou não invasivas, como educações posturais e conscientização corporal; terapia manual, como por exemplo, a compressão isquêmica, exercícios, alongamentos, entre outros; e a eletrofototerapia, que inclui o ondas de choque, o ultrassom terapêutico, a laserterapia, entre outros (BORG-STEIN; IACCARINO, 2014; GALASSO et al., 2020; RICKARDS,2006).O Agulhamento a Seco (AS), também conhecido como Dry Needling, consiste na perfuração da pele, tecidos superficiais e musculares utilizando uma agulha fina. Com a aplicação da agulha, há microlesão que vai ativar três mecanismos, o neurofisiológico, o químico e o mecânico, que resultará em redução da hipersensibilidade à dor, o alongamento dos sarcômeros, homeostase química local e melhora da circulação próxima ao PG. Além disso, ocorre

o aumento do limiar de dor por pressão e a melhora da função muscular. (GATTIE; CLELAND; SNODGRASS, 2017;

ZIAEIFAR et al., 2013; CAGNIE et al., 2012; DOMMERHOLT, 2004).No momento que há a inserção da agulha no PG, ocorre uma vasodilatação e

aumento de fluxo sanguíneo local, que resulta em diminuição de substâncias algogênicas, presentes na formação dos PG, além da redução de ativação dos nociceptores. Efeitos adversos que podem ocorrer com a aplicação da agulha são reprodução e/ou aumento momentâneo da dor, perfuração de pequenos vasos com eventual sangramento, formação de pequenas equimoses e reação de hiperemia local (CARVALHO et al.,

2017).A compressão isquêmica (CI) é um tratamento utilizado para a liberação do PG, consiste na aplicação de pressão sob o PG até que o desconforto diminua. Pode ser incrementada maior pressão até a completa liberação do PG. Ela ocasiona o alongamento dos sarcômeros, melhora do fluxo sanguíneo retirando substâncias químicas nocivas que resulta na redução da tensão muscular local, aumento da ADM, alívio da dor e aumento do limiar de dor por pressão (GRIEVE et al., 2013; RICKARDS, 2006; SIMONS, 2004; TABATABAIEE et al., 2019). A terapia por ondas de choque (TOC) é executada por um equipamento que emite uma onda sonora de alta energia produzida pela liberação de alta pressão de ar. Essa energia mecânica se propaga no tecido e ocasiona microlesões, microrrupturas dos capilares e de mediadores químicos por meio do mecanismo de cavitação, com isso, o resultado é a melhora na revascularização e na regeneração tecidual, diminuição da inflamação, analgesia, além da liberação dos PG, levando em conta que a onda acústica desbloqueia a bomba de cálcio diminuindo o ciclo de contração constante (KIRÁLY; BENDER; HODOSI, 2018; KISCH et al., 2016; PARK et al., 2018; TAHRIRIAN et al., 2012; WANG; WANG; YANG, 2004).As terapias de AS, CI e a TOC são algumas das terapias que são encontradas na literatura, no entanto ainda existem lacunas a respeito dos melhores recursos e parâmetros para o tratamento de pontos gatilhos miofasciais. Assim, o objetivo deste estudo será comparar os efeitos de uma sessão de tratamento de pontos gatilhos miofasciais com AS, CI e TOC após 1 sessão e após 48 horas nos níveis de dor, temperatura corporal, exame de imagem e força.

Objetivo da Pesquisa:

Objetivo Primário:

Comparar os efeitos agudos e crônicos de três técnicas no tratamento de pontos gatilhos miofasciais.

Objetivo Secundário:
Analisar os efeitos da compressão isquêmica no PG;
Analisar os efeitos da terapia por ondas de choque no PG;
Analisar os efeitos do dry needling noPG;
Verificar o efeito das técnicas nas variáveis de dor, força, amplitude de movimento, atividade elétrica e desempenho (função) do segmento
avaliado. Verificar a concordância entre as imagens da termografia e da ultrassonografia com a palpação digital dos pontos gatilhos.
Verificar relação das terapias com a força da musculatura e dor do PG;

Avaliação dos Riscos e Benefícios:
Riscos:
Por se tratar de uma condição muscular, cujo tratamento baseia-se em microlesões do ponto gatilho ou nódulo de tensão, é possível que ocorra dor muscular no momento da aplicação da técnica e algumas horas após. No entanto, apesar do desconforto previsto, nenhuma das intervenções geram riscos de danos relevantes conhecidos para a função ou estrutura muscular.
Benefícios:
Os benefícios diretos previstos ao sujeito da pesquisa dizem respeito a melhora da função muscular após a aplicação das técnicas de tratamento,
tendo em vista que todas são comprovadamente eficazes, causando analgesia, melhora da circulação local e da função muscular. Os benefícios indiretos dizem respeito ao maior conhecimento a respeito das técnicas utilizadas para o tratamento dos pontos gatilhos miofasciais, auxiliando no maior conhecimento científico aplicado a prática clínica.

Comentários e Considerações sobre a Pesquisa:
Trata-se pesquisa com tema relevante, com desenho metodológico bem estruturado .

Considerações sobre os Termos de apresentação obrigatória:
Termos obrigatórios adequados.

Recomendações:
Não há recomendações.

Conclusões ou Pendências e Lista de Inadequações:
Considerando a documentação apresentada, o projeto pode ser considerado aprovado.

Considerações Finais a critério do CEP:
O CEP da FFC da UNESP de MARÍLIA, em reunião ordinária de 19/05/2021, após acatar o parecer

Endereço: Av. Hygino Muzzi Filho, 737
Bairro: Campus Universitário **CEP:** 17.525-900
UF: SP **Município:** MARILIA
Telefone: (14)3402-1346 **E-mail:** cep.marilia@unesp.br

do membro relator previamente aprovado para o presente estudo e atendendo a todos os dispositivos das resoluções 466/2012, 510/2016 e complementares, bem como ter aprovado o Termo de Consentimento Livre e Esclarecido como também todos os anexos incluídos na pesquisa, resolve APROVAR a pesquisa "AVALIAÇÃO DE DIFERENTES MÉTODOS UTILIZADOS NO TRATAMENTOS NOS PONTOS GATILHOS MIOFASCIAIS: ENSAIO CONTROLADO ALEATORIZADO".

Este parecer foi elaborado baseado nos documentos abaixo relacionados:

Tipo Documento	Arquivo	Postagem	Autor	Situação
Informações Básicas do Projeto	PB_INFORMAÇÕES_BÁSICAS_DO_PROJETO_1751318.pdf	10/05/2021 20:58:42		Aceito
Declaração de Instituição e Infraestrutura	AutorizacaoPesquisa.pdf	10/05/2021 20:52:29	CRISTIANE RODRIGUES PEDRONI	Aceito
Projeto Detalhado / Brochura Investigador	Projeto.pdf	10/05/2021 20:51:58	CRISTIANE RODRIGUES PEDRONI	Aceito
TCLE / Termos de Assentimento / Justificativa de Ausência	TCLE.pdf	10/05/2021 20:49:36	CRISTIANE RODRIGUES PEDRONI	Aceito
Folha de Rosto	FolhaRosto.pdf	10/05/2021 20:48:52	CRISTIANE RODRIGUES PEDRONI	Aceito

Situação do Parecer:
Aprovado

Necessita Apreciação da CONEP:
Não

MARILIA, 09 de Junho de 2021

Assinado por:
SIMONE APARECIDA CAPELLINI
(Coordenador(a))

Endereço: Av. Hygino Muzzi Filho, 737
Bairro: Campus Universitário **CEP:** 17.525-900
UF: SP **Município:** MARILIA
Telefone: (14)3402-1346 **E-mail:** cep.marilia@unesp.br
